# Causes of Unwarranted Variation and Disparity in Breast Cancer Management in Regional and Rural Area

**DOI:** 10.1155/2024/9354395

**Published:** 2024-06-19

**Authors:** Kimberley J. Davis, Chantal Campbell, Rebekah Costelloe, Ting Song, Glaucia Fylyk, Ping Yu, Steven J. Craig

**Affiliations:** ^1^Research Operations, Illawarra Shoalhaven Local Health District, Wollongong, NSW, Australia; ^2^Graduate School of Medicine, Faculty of Science Medicine and Health, University of Wollongong, Wollongong, NSW, Australia; ^3^Illawarra Shoalhaven Cancer and Haematology Network, Illawarra Shoalhaven Local Health District, Wollongong, NSW, Australia; ^4^Centre for Digital Transformation, School of Computing and Information Technology, Faculty of Engineering and Information Sciences, University of Wollongong, Wollongong, NSW, Australia; ^5^Illawarra Cancer Care Centre, Wollongong Hospital, Wollongong, NSW, Australia; ^6^Department of Surgery, Shoalhaven District Memorial Hospital, Nowra, NSW, Australia

## Abstract

**Introduction:**

Breast cancer management is complex, requiring personalised care from multidisciplinary teams. Research shows that there is unwarranted clinical variation in mastectomy rates between rural and metropolitan patients; that is, variation in treatment which cannot be explained by disease progression or medical necessity. This study aims to determine the clinical and nonclinical factors contributing to any unwarranted variation in breast cancer management in rural patients and to evaluate how these factors and variations relate to patient outcomes.

**Methods:**

Comprehensive data from patients who had primary breast cancer surgery from 2010 to 2014 in either a rural or metropolitan location in a single local health district was analysed (*n* = 686). Records were subset into two rurality groupings based on the postcode in which the patient resided, and the Modified Monash Model (MMM), an Australian system for classifying rurality. Statistical analysis was used to compare rural and metropolitan cohorts on treatments, patient characteristics, timeliness, and outcomes (recurrence and survival).

**Results:**

Rural patients had higher mastectomy rates than metropolitan patients (57% vs. 34%, *p* < 0.001), despite a lack of difference in clinical or demographic factors accounting for such variation. The length of time between treatment pathway stages was consistently longer amongst rural patients (*p* < 0.01). Rural women also had worse survival outcomes, especially amongst HER2-positive patients who had significantly lower survival (5-year 74% vs 82%; 10-year 49% vs 71%, *p* < 0.05) than metropolitan HER2-positive patients.

**Conclusion:**

This study reveals clinical disparities among rural breast cancer patients, that cannot be explained by demographic and clinical factors alone. Rural patients face lower rates of breast-conserving surgery and treatment delays, attributable to systemic barriers such as limited access to specialist care, high travel costs, and suboptimal care coordination. These findings have important implications for improving equity and collaboration in delivering person-centred breast cancer care.

## 1. Introduction

Breast cancer is the most commonly diagnosed cancer in Australia and the second most common cause of cancer-related death among women [1]. The introduction of mammographic screening (BreastScreen Australia) and advances in local and systemic therapies have led to a significant reduction in national breast cancer mortality rates in recent decades [1]. However, variation in breast cancer treatment and outcomes between women in rural areas and metropolitan areas still exists [2]. Women with breast cancer living in remote areas experience poorer survival outcomes than those in metropolitan areas, despite comparable staging [1, 3, 4]. Such disparities also exist among other groups, such as those with lower socioeconomic status.

In addition to poorer survival measures, rural breast cancer patients often receive different treatment to their metropolitan counterparts. For instance, studies consistently show lower rates of breast conserving surgery (BCS) among rural women compared to those in urban areas despite similar staging and patient features [2, 5, 6]. One study showed that rural breast cancer patients are at least five times more likely to undergo mastectomy compared to metropolitan women [4], indicating the presence of unwarranted clinical variation in breast cancer management, which cannot be accounted for by disease progression or medical need for a mastectomy. The causes, however, are likely multifactorial given the many stages involved in optimal breast cancer management. While the issue of unwarranted variation in breast cancer management is evident in Australia, similar disparities are observed globally. Studies from broadly comparable countries such as Canada, the UK, and the United States also demonstrate variations in treatment and outcomes based on geographic location and access to healthcare services [7–9].

Breast cancer management is complex and involves a multidisciplinary approach in which a number of healthcare professionals collaborate to create personalised treatment plans within standardised guidelines. The *Optimal Care Pathway* is an Australia-wide breast cancer care model that outlines seven critical stages in a patient's journey: prevention, presentation, diagnosis, treatment, post-treatment care, recurrence management, and end-of-life considerations [10]. Although these steps are presented linearly, patient treatment decisions may vary based on numerous clinical features such as age, tumour characteristics (size, stage, and receptor status), and patient comorbidities, as well as nonclinical factors such as access to specialist services and availability of adjuvant therapies [11]. Rural patients face significant barriers to accessing healthcare services at all levels, including cancer screening, primary care, and specialised oncology facilities. Inaccessibility of health services may result in delays in diagnosis and suboptimal treatment at all stages, from screening to post-treatment care [3, 12, 13].

There is a need for comprehensive exploration into the underlying contributing factors of the unwarranted clinical variation in breast cancer treatment between rural and metropolitan women to ensure all women receive the best available treatments and outcomes. In Australia, breast cancer data is managed by state Cancer Institutes, which report on the management of breast cancer across different hospital and district health services, and often identify clear and unwarranted variation in breast cancer management [14]. However, whilst these reports are valuable for identifying outlying treatment variations in regional areas, they do not explore the reasons behind such discrepancies.

The aim of this study is to investigate breast cancer management in a single health district to determine if there was any difference in management and outcomes between regional and metropolitan patients, and then to determine the clinical and nonclinical factors which may contribute to any unwarranted variation in breast cancer management.

## 2. Methods

### 2.1. Setting

The health district selected for study was the Illawarra Shoalhaven Local Health District (ISLHD), located on the New South Wales (NSW) south coast of Australia. This location was chosen due to its unique geographical characteristics; in particular, it has a large metropolitan centre and tertiary hospital that is within 1.5 hours' drive of smaller rural/regional town with a smaller secondary hospital. Both sites are governed by the same Local Health District, feed into the same multidisciplinary team (MDT) meeting, and have the same investigation modalities and adjuvant therapies available. Any difference observed in the treatment pathways or outcomes would then be less likely to be the result of variation between service providers and more reflective of unique geographic factors.

### 2.2. Data

Records of patients who received a breast-related surgery between January 2010 and December 2014 at either Wollongong Hospital (metropolitan) or Shoalhaven District Memorial Hospital (rural/regional) were acquired from the Illawarra Shoalhaven Local Health District's (ISLHD) SurgiNet database (*n* = 1,040). These records were then correlated with medical and radiation oncology data held within ISLHD's MOSAIQ Oncology Information System in addition to the NSW Cancer Registry [15].

Records for inclusion were those relating to a primary breast cancer diagnosis (C50.0–C50.9) between 2010 and 2014 (inclusive), with their surgery performed at an ISLHD hospital. Records were evaluated individually, and exclusions were made based upon the following criteria:

1. Ductal Carcinoma In Situ (D05.0, D05.1, D05.7, or D05.9)
2. Unrelated or benign finding (*e.g.* N62 Hypertrophy of breast, D24 Benign neoplasm of breast, N60.2 Fibroadenosis of breast etc.)
3. A breast cancer diagnosis within the study period which was a recurrence of an earlier primary prior to the study period
4. Surgeries which were prophylactic only and not related to a breast cancer event (Z40.0 or Z40.8)
5. Secondary breast cancer (*e.g.* C79.81 secondary malignant neoplasm of breast)
6. Lymph node surgery only (e.g. R59.1 Generalised Enlarge lymph nodes or C77.3 Secondary and unspecified malignant neoplasm of axillary and upper limb lymph nodes)
7. Phyllodes tumour
8. Patients who were given neo-adjuvant hormone therapy
9. Primary surgery discovered to be at a non-ISLHD hospital

A total of 686 records remained after exclusions, which were then subset into two rurality groupings based on the postcode in which the patient resided and the Modified Monash Model (MMM), an Australian classification system for rurality [16, 17]. MMM categories range from MM1 (major city) to MM7 (very remote); it takes into account population sizes in addition to geographical remoteness and is used for health workforce planning within rural and remote areas [17]. Records within categories MM1 (metropolitan areas) and MM2 (Regional Centres) were grouped (*n* = 492), and hereafter are referred to as “Metro;” MM3 (large rural towns), MM4 (medium rural towns), and MM5 (small rural towns) were grouped (*n* = 194) and are hereafter referred to as “Rural.” There were no patients from MM6 (remote communities) or MM7 (very remote communities) localities.

A range of patient and clinical factors were then evaluated to assess for unwarranted clinical variation in breast cancer surgeries and treatment pathways between rural and metropolitan patients. A comprehensive list of data variables, either retrieved or calculated appear in Supplementary Table 1. Treatment milestone times were additionally calculated as the elapsed time between diagnosis date (biopsy) and each relevant treatment milestone.

### 2.3. Statistical Analysis

All statistical evaluation was conducted using R and R Studio (version 3.6.3) [18], and the following packages were used for data management and statistical tests: tidyverse (v1.3.1) [19], gtsummary (v1.7.1) [20], janitor (V2.1.0) [21], rstatix (v0.7.2) [22], epitools (v0.5-10.1) [22], survival (v3-5.3) [23], and ggsurvfit (v0.2.1) [24]. Descriptive statistics (means, standard deviations, and proportions) were initially used to assess the data. Chi-squared tests of association were used to evaluate differences in proportions, and Wilcoxon rank sum (Mann–Whitney *U*) tests were used to evaluate differences in continuous data against categorical variables; phi-coefficients (*φ*) were calculated according to Yule [25], and the magnitude of association (|*r*|) was determined according to Cohen's benchmarks [26]. Kaplan–Meier curves were constructed for survival data, and differences in survival curves evaluated using log-rank tests plus cox hazard ratios. Binomial logistic regression was used to evaluate predictors of binary outcomes.

## 3. Statement of Ethics

This study was approved by the Joint University of Wollongong and Illawarra Shoalhaven Local Health District Human Research Ethics Committee (2021/ETH00525).

## 4. Results

### 4.1. Surgery Type

**Figure 1:**
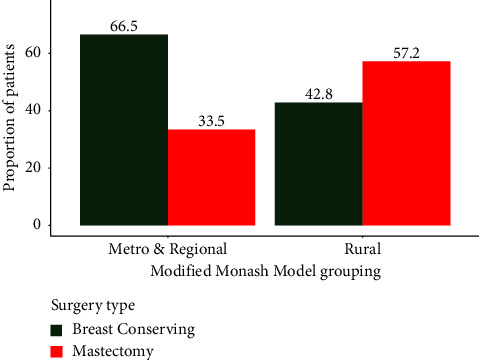
Proportion of patients having either mastectomy or breast conserving surgery according to their Modified Monash Model grouping.

There were 686 records of patients with a primary breast cancer diagnosis within the study period. Of these, 492 were from Metro areas and 194 from Rural areas. Cohort descriptive statistics appear in Table 1. There was no statistically significant difference in age, proportion of indigenous patients, or the average number of surgeries between metro and rural patients, though rural patients had a higher average number of comorbidities (3.7 vs 4.2, *p*<0.01). There was, however, a difference in the initial procedure performed (Figure 1), with just over half (*n* = 111, 57%) of rural patients undergoing a mastectomy, compared to only a third of metro patients (*n* = 165, 34%), which was statistically significant and of strong association (*χ*^2^ = 31.5, *p* < 0.001, *φ* = 0.214). Odds ratio calculations showed that rural patients were over two and a half times more likely to have an initial mastectomy compared to their metro counterparts (OR = 2.65, 95% CI [1.88–3.72], *p* < 0.001). After accounting for all surgeries performed (*e.g.* surgical margins not clear and re-excision required), rural patients were still more likely and strongly associated with having a mastectomy than metro patients (Table 1: *χ*^2^ = 28.4, *φ* = 0.192, OR = 2.40, 95% CI [1.71–3.38], *p* < 0.001). There was a higher proportion of metro patients requiring more than one surgery; however, the length of time taken for completion of all surgeries was greater amongst rural patients (33 vs 24 days, *U* = 348, *r* = 0.315, *p* < 0.01) and of moderate effect size. Rural patients had a significantly longer average length of stay (LOS) than metro patients (*p* < 0.001), reflective of the higher number of mastectomies performed on rural patients, which typically have a longer LOS than breast conserving surgeries.

To account for the differences in surgery type according to patient rurality, a range of clinical factors were examined (Table 2). There were no statistically significant differences between TNM staging, histopathological grade, tumour size, receptor status [27], or screening rate. Rural patients however were more likely to be within the BreastScreen Australia targeted screening age group (50–74) [28] (*χ*^2^ = 9.50, *φ* = 0.118, OR = 1.76, 95% CI [1.24–2.51], *p* < 0.01), and the association was strong.

Binomial logistic regression models with a range of variables did not reveal any factors that could predict a mastectomy over rurality (Table 3); age, screening rate, tumour size, staging, and histopathological grade were all associated with mastectomy to some extent (all *p* < 0.001). When each was added as covariates to the model, the odds of mastectomy for rural patients increased slightly. This result demonstrates that patient rurality is the strongest predictor of mastectomy, more than any other clinical deature or demographic characteristic evaluated. Conversely, the odds of a mastectomy decreased slightly with receptor status as a covariate, but this change was minimal. For receptor status, this was mainly reflected in slightly higher odds of HER2-positive patients having a mastectomy, of which there were slightly more in rural areas (Table 2).

### 4.2. Treatment Pathway Timing

In an effort to further investigate variations in clinical management pathways beyond the factors discussed above, the timeliness of treatment access was investigated.  Table 4 shows the average number of weeks taken to reach each of these per rurality group. Every milestone except time to TNM staging was statistically significantly different (*p* < 0.01, small to moderate effect sizes), and in each instance rural patients experienced a longer wait time than their metro counterparts. Even when the multiple adjuvant therapy pathways were evaluated separately (*i.e.* radiotherapy alone, chemotherapy/immunotherapy alone, both, or neither), rural patients consistently experienced longer wait times for each step of their clinical management pathway (Supplementary Table 2).

### 4.3. Patient Outcomes

A range of outcomes were evaluated to assess the impact of these discrepancies (Table 5). Overall, rural patients had slightly worse outcomes than metro patients, in terms of both years of survived and recurrence (being either locoregional recurrence or distant metastases); however, these differences were not statistically significant.

There were however disparities in outcomes for metro and rural patients when stratified by receptor status; specifically, rural patients with HER2-positive tumours (*n* = 28) fared worse in terms of overall length of survival (7.9 vs 9.7 years, *U* = 935, *p* < 0.05, *r* = 0.255), and were nearly three times more likely to die (OR = 2.87, 95% CI [1.08-7.82], *p* < 0.05) compared to their metro counterparts (Table 6). Although rates of recurrence or metastasis amongst rural patients were around three times that of metro patients, this difference was just outside statistical significance, likely due to the small sample size of this subgroup. No significant differences were found amongst any other receptor subtypes.

## 5. Discussion

Results from this study clearly show disparities in the clinical management of and outcomes for rural patients diagnosed with breast cancer. The initial hypotheses were that the higher rates of mastectomy amongst rural patients were due to common perceptions that rural patients were older, had lower participation in routine screening, or presented later with higher grade, stage, and/or larger tumours, any of which may warrant a mastectomy over BCS. However, detailed statistical evaluation of these factors showed that rural patients have similar demographic and tumour features to metro patients (Tables 1 and 2), yet have a significantly lower rate of BCS. These findings are consistent with a 2018 systematic review which evaluated the disparities in breast cancer treatment and outcomes by geographical location. Of the 13 studies included, eight found no difference in tumour characteristics between metropolitan and nonmetropolitan women; there was an evenly-spread mix in terms of differences in screening rate; and six out of eight studies found higher mastectomy rates amongst rural women [4]. The mastectomy rates in the present study are also consistent with those reported in an earlier South Australian study [29]. Nevertheless, reasons for the variation observed in this study still remain unclear. Other potential factors that could not be captured in this study might include community attitudes to BCS [30, 31], or surgeon education and training.

Another telling disparity in access to care for rural patients were the differences in time to each treatment pathway milestone (Table 4). Rural patients consistently waited longer than metro patients at each step, but the reasons for this disparity are unclear. Importantly though, the time waited until the initial surgery is a key rate-limiting factor in determining the overall timeframe for a patient's treatment, as many subsequent steps rely on the completion of surgery in order to progress. Timeliness of treatment is a key factor in equitable access to cancer care for rural Australians. Delays are often ascribed to the burden and cost of travel, in addition to factors such as a lack of access to primary and specialist care, higher out-of-pocket costs, and poorer coordination and continuity of care [4, 32, 33]. In the absence of patient-reported information and data on their socioeconomic status, it is difficult to pinpoint the exact causes of the delays identified in this study, despite them all being plausible explanations. Previous work has shown that referral pathways for rural women were often delayed, particularly with respect to specialist assessment and subsequent treatment [32, 34, 35]. Despite the development and implementation of measures such as Optimal Care Pathways [10], accurately implementing these measures in a rural setting still appears to be challenging.

The impact of the disparities identified in this study was evaluated in terms of outcomes such as death, local recurrence, and distant metastases. Although not statistically significant, rural patients experienced higher rates of all three outcomes, with the largest disparity being amongst rates of distant metastases (8.3% for metro vs 12% for rural). Research has identified poorer survival outcomes for rural cancer patients in general, including those with breast cancer [4]. We identified a significant survival difference amongst rural HER2-positive patients, which were not observed in any other cancer subtype. However, in the absence of any discernible demographic or clinical differences amongst this group, there is no obvious explanation. Higher-level investigations of the pathway taken by these patients are required.

This study has several limitations. Primarily, the retrospective and historical nature of the data means that there are restrictions on the amount of information available, including access to any patient-reported information which may explain the results observed. There have also been changes in treatment patterns since the study period, meaning some patient pathways valid at the time are no longer so by current standards. Another limitation was the absence of detailed socioeconomic information beyond postcode (which was used to determine rurality), which may potentially influence the study results. Furthermore, although overall disease-free survival information is useful it could not be accessed due to governance constraints. Finally, there is a reliance on coding within the data, such as for designation of diagnoses as primary or recurrence, which may cause error in assignment of a small number of records.

A strength of this study is the ability to compare pathways for rural and metro patients who received their treatment within one health service (ISLHD). This removes confounders such as differences in local processes between health services which may contribute to perceived disparities. Furthermore, although differences in optimal management are regularly reported at a State-level [14], this study, to the best of our knowledge, represents the first detailed evaluation of a range of potential clinical, demographic, and geographic causes for the observed disparities. Finally, the comprehensive dataset evaluated in this study are eminently amenable to process mining and machine learning techniques, methods which offer unique insights beyond traditional statistical approaches. This evaluation is already underway and will enable detailed linkage of observed patient pathways with outcomes.

## 6. Conclusions

Rural patients are at much higher risk of receiving mastectomy over BCS than their metropolitan counterparts, despite having similar clinical and demographic indicators for this surgery. They also wait longer for nearly every step of their treatment pathway and have marginally poorer outcomes in terms of overall survival and recurrence or metastases. Disparities in these outcomes are more apparent amongst HER2-positive patients.

Clearly, there are factors at play for the unwarranted variation in clinical practice within the study cohort, which are beyond the reach of this retrospective secondary data analytics study. The existing literature points to a range of interdependent contributing factors, meaning that further work is required to fully understand the reasons for disparities observed within this cohort. This may include detailed socioeconomic and geospatial analysis, plus qualitative exploration of patient preference and perceived barriers to care. While patient preference is an important factor, its evaluation must also consider the information conveyed during the surgeon's patient education. Furthermore, the complexity of breast cancer pathway options—although clinically warranted according to subtypes and histopathological factors—makes a regular statistical analysis of the data somewhat one-dimensional and not sensitive enough to detect the nuances of cancer treatment. More advanced tools such as process mining or machine learning may prove useful in this area.

## Figures and Tables

**Table 1:** Cohort descriptive statistics for each rurality group.

Variable Mean (SD) or *n* (%)	Metro and regional	Rural	*p* value	Statistic
Age (years)	64.3 (13.6)	63.8 (12.0)	0.4	—
Indigenous status			0.9	—
Aboriginal but not Torres strait islander origin	14 (2.8%)	4 (2.0%)		
Neither aboriginal nor Torres strait islander origin	477 (97%)	190 (98%)		
Not stated	2 (0.2%)	0 (0%)		
Number of comorbidities	3.7 (2.2)	4.2 (2.5)	**0.006**	*U* = 41424 *r* = 0.105 (small)
Number of surgeries			0.076	—
1	418 (85%)	177 (91%)		
2	70 (14%)	16 (8.2%)		
3	4 (0.8%)	1 (0.5%)		
Time between first and last surgeries (days)	24 (20)	33 (15)	**0.003**	*U* = 348 *r* = 0.315 (medium)
Initial procedure			**<0.001**	*χ* ^2^ = 31.5 *φ* = 0.214 (strong)
Breast conserving	327 (66%)	83 (43%)		
Mastectomy	165 (34%)	111 (57%)		
Eventual procedure			**<0.001**	*χ* ^2^ = 25.4 *φ* = 0.192 (strong)
Breast conserving	304 (62%)	78 (40%)		
Mastectomy	188 (38%)	116 (60%)		
Initial LOS (days)	2.1 (2.1)	3.4 (3.0)	**<0.001**	*U* = 34169 *r* = 0.242 (medium)

*P* values less than 0.05 were considered statistically significant as stated bold values.

**Table 2:** Clinical factors for each rurality group.

Variable *n* (%)	Metro and regional	Rural	*p* value	Statistic
TNM stage			0.10	—
I	228 (46%)	78 (40%)		
II	205 (42%)	87 (45%)		
III	56 (11%)	24 (12%)		
IV	3 (0.6%)	5 (2.6%)		
Tumour size (mm)	22.8 (17.1)	24.4 (21.6)	0.6	—
Histopathological grade			0.6	—
1	94 (19%)	39 (20%)		
2	233 (48%)	82 (43%)		
3	158 (32%)	71 (37%)		
9	2 (0.4%)	1 (0.5%)		
Receptor status			0.4	—
HER2 positive	51 (11%)	28 (15%)		
Luminal A	341 (71%)	125 (67%)		
Luminal B	46 (9.5%)	15 (8.0%)		
Triple negative	46 (9.5%)	19 (10%)		
Initial presentation			0.2	—
Screening	204 (42%)	83 (48%)		
Symptomatic	279 (48%)	89 (52%)		
Screening age (50–74)			**0.002**	*χ* ^2^ = 9.50 *φ* = 0.118 (strong)
Non-screening age	217 (44%)	60 (31%)		
Screening age	275 (56%)	134 (69%)		

*P* values less than 0.05 were considered statistically significant as stated bold values.

**Table 3:** Binomial logistic regression models predicting incidence of mastectomy.

	Odds ratio	95% CI	*p* value
Metro	—^a^	—	**<0.001**
Rural	2.65	1.89–3.73	
Age	1.02	1.01–1.04	**<0.001**
Grade 1	—^a^	—	**=0.002**
Grade 2	2.11	1.34–3.38	**<0.001**
Grade 3	3.50	2.19–5.71	0.7
Grade 9	1.65	0.07–17.7	
Luminal A	—^a^	—	
Luminal B	1.18	0.68–2.03	0.5
HER2 positive	3.72	2.26–6.25	**<0.001**
Triple negative	1.22	0.71–2.06	0.5
Stage I	—^a^	—	
Stage II	3.63	2.55–5.22	**<0.001**
Stage III|	13.0	7.34–24.1	**<0.001**
Stage IV	26.5	4.60–499	**=0.002**
Screening	—^a^	—	**<0.001**
Symptomatic	2.689	1.92–3.75	
Size	1.04	1.03–1.06	**<0.001**

*Rurality with covariates*			

Metro	—^a^	—	
Rural	2.75	1.95–3.90	**<0.001**
Age	1.03	1.01–1.04	**<0.001**
Metro	—^a^	—	
Rural	2.81	1.98–4.01	**<0.001**
Grade 1	—^a^	—	
Grade 2	2.28	1.43–3.70	**<0.01**
Grade 3	3.68	2.27–6.09	**<0.001**
Grade 9	1.61	0.07–18.5	0.7
Metro	—^a^	—	
Rural	2.64	1.86–3.77	**<0.001**
Luminal A	—^a^	—	
Luminal B	1.22	0.69–2.13	0.5
HER2 positive	3.64	2.19–6.18	**<0.001**
Triple negative	1.20	0.69–2.05	0.5
Metro	—^a^	—	
Rural	2.81	1.94–4.09	**<0.001**
Stage I	—^a^	—	
Stage II	3.70	2.57–5.37	**<0.001**
Stage III	14.0	7.79–26.3	**<0.001**
Stage IV	21.1	3.50–404	**=0.005**
Metro	—^a^	—	
Rural	2.98	2.06–4.35	**<0.001**
Screening	—^a^	—	
Symptomatic	3.00	2.13–4.27	**<0.001**
Metro	—^a^	—	
Rural	2.86	2.00–4.12	**<0.001**
Size	1.05	1.03–1.06	**<0.001**

^a^Reference levels. *P* values less than 0.05 were considered statistically significant as stated bold values.

**Table 4:** Time in weeks between diagnosis and each clinical pathway milestone by rurality.

Time to milestone (weeks)	Metro and regional	Rural	Metro and regional	Rural	*p* value	Statistic
	Mean (SD)		Median (IQR)			
Surgery	2.7 (1.6)	3.6 (1.8)	2.3 (1.6)	3.3 (2.3)	**<0.001**	*U* = 30868 *r* = 0.276 (medium)
Final surgery	3.2 (2.3)	3.6 (1.8)	2.4 (2.0)	3.6 (2.7)	**<0.001**	*U* = 34950 *r* = 0.209 (medium)
Oncologist referral	5.6 (3.9)	7.2 (3.6)	4.9 (2.9)	6.9 (3.7)	**<0.001**	*U* = 19304 *r* = 0.287 (medium)
Oncologist consultation	7.9 (3.9)	8.7 (4.1)	7.3 (3.3)	7.9 (3.9)	**0.010**	*U* = 32516 *r* = 0.104 (small)
TNM staging	7.5 (5.8)	8.3 (6.2)	6.9 (4.0)	7.1 (4.1)	0.2	—
MDT	5.3 (7.7)	6.8 (2.7)	4.4 (2.6)	6.4 (2.6)	**<0.001**	*U* = 24526 *r* = 0.370 (medium)
Chemotherapy or systemic therapy	10.5 (5.8)	11.7 (3.5)	9.5 (3.2)	11.5 (4.3)	**<0.001**	*U* = 4126 *r* = 0.276 (medium)
Radiation therapy	20.8 (10.3)	24.2 (11.0)	16.9 (15.8)	22.0 (17.1)	**0.003**	*U* = 12202 *r* = 0.149 (small)

*P* values less than 0.05 were considered statistically significant as stated bold values.

**Table 5:** Survival and recurrence outcomes by rurality.

Outcome Mean (SD) or *n* (%)	Metro and regional	Rural	*p* value
Overall survival (years)	9.6 (3.4)	9.3 (3.5)	0.3
Survival likelihood			
5-year	86%	84%	0.3
10-year	72%	69%	
Deceased	151 (31%)	67 (35%)	0.3
Recurrence or metastases (combined)	54 (11%)	30 (15%)	0.11
Local recurrence	13 (2.6%)	6 (3.1%)	0.7
Distant metastases	41 (8.3%)	24 (12%)	0.10

**Table 6:** Survival and recurrence outcomes by rurality for HER2-positive subtypes.

Outcome Mean (SD) or *n* (%)	Metro and regional	Rural	*p* value	
Overall survival (years)	9.7 (3.6)	7.9 (3.9)	**0.024**	*U* = 935 *r* = 0.255 (medium)
Survival likelihood				
5-year	82%	74%	**0.029**	*β* = 0.821
10-year	71%	49%		HR = 2.27 [1.07-4.84]
Deceased	13 (25%)	14 (50%)	**0.028**	*χ* ^2^ = 3.80 *φ* = 0.219 (medium)
Recurrence or metastases (combined)	5 (9.8%)	8 (29%)	0.054	—

*P* values less than 0.05 were considered statistically significant as stated bold values.

## Data Availability

The data that support the findings of this study are available on request from the corresponding author. The data are not publicly available due to privacy and ethical restrictions.

## Supplementary Materials

Supplementary Materials Supplementary Table 1—A full list and description of data variables utilised in this study. Supplementary Table 2—Time in weeks between diagnosis and each clinical pathway milestone by rurality for each adjuvant treatment regimen subgroup (radiotherapy alone, chemotherapy alone, both radiotherapy and chemotherapy, plus no adjuvant therapy).
