# Supplementary material for: Causes of Unwarranted Variation and Disparity in Breast Cancer Management in Regional and Rural Area
Source: Breast J. 2024 Jun 19;2024:9354395. doi: 10.1155/2024/9354395 (PMC11208097; doi:10.1155/2024/9354395)
Supplement: Supplementary Materials — Supplementary Table 1—A full list and description of data variables utilised in this study. Supplementary Table 2—Time in weeks between diagnosis and each clinical pathway milestone by rurality for each adjuvant treatment regimen subgroup (radiotherapy alone, chemotherapy alone, both radiotherapy and chemotherapy, plus no adjuvant therapy). [file 9354395.f1.docx]

# Supplementary Material

**Supplementary Table 1 – Data variables**

| **Variable** | **Description** |
| --- | --- |
| *Retrieved fields* | |
| Surgery Facility | P208 = Wollongong Hospital  P207 = Shoalhaven District Memorial Hospital |
| Age at surgery | - |
| Postcode | Patient’s postcode of residence at the time of their surgery |
| Indigenous Status | Aboriginal  Aboriginal but not Torres Strait Islander  Neither Aboriginal nor Torres Strait Islander  Not Stated |
| Admission / Discharge date and time | - |
| Length of stay | Recorded in whole days |
| Procedure code and description | ICD10 Version 11 |
| Diagnosis code and description (Surgery) | ICD10 Version 11 |
| Procedural consultant | - |
| Primary, planned and actual procedures | - |
| Primary Diagnosis Site (Oncology) | ICD10 Version 11 |
| Diagnosis date | Date of biopsy (or closest evidence of diagnosis where biopsy date not recorded) |
| Oncologist referral date | First known date where a patient was referred to either a medical or radiation oncologist |
| Oncologist consultation date | First date a patient received a consultation with either a medical or radiation oncologist |
| Morphology code | Morphology code from the histopathological report |
| Laterality | Laterality of primary breast cancer (Left/Right) |
| Histopathological Grade | Nottingham Score for Breast Cancer  Grade I – well differentiated  Grade II – moderately differentiated  Grade III – poorly differentiated |
| TNM Staging plus date of staging | Pathological staging information was used as a first preference; where this was not available or had not been completed, clinical staging was used |
| MDT date | Date of the first multidisciplinary team meeting where a case was discussed |
| ER, PR and HER2 status | - |
| Radiation Therapy start and end date, course name, prescribed and actual dose, prescribed and actual fractions | - |
| Chemotherapy start and end date plus protocol | Chemotherapy also includes other systemic therapy such as Herceptin |
| Hormonal therapy start date and type of therapy | - |
| Date of death | - |
| Initial Presentation | Screening, Symptomatic, other or not recorded |
| Tumour Size (mm) | - |
| *Calculated fields* | |
| Number of diagnoses | Number of ICD10 diagnosis codes present per patient |
| Number of operations | Number of breast operations performed |
| Recurrence or metastasis | Binary Y/N  Where multiple breast cancer diagnoses appeared, each diagnosis was evaluated and categorised as either a recurrence/ metastasis, or a new primary cancer. |
| Local Recurrence | Binary Y/N |
| Distant Metastasis | Binary Y/N |
| Receptor status[24] | Luminal A = ER+, PR+, HER2-  Luminal B = ER+, PR-, HER2-  HER2 Positive = ER-, PR-, HER2+  TNBC = ER-, PR-, HER2- |
| Chemotherapy timing | Adjuvant, Neoadjuvant or No Chemotherapy |
| Treatment regimen | Chemotherapy only, radiation only, both or neither |
| Rurality | Modified Monash Model according to postcode of residence. |
| Survival since diagnosis | For deceased patients, the difference between diagnosis date and date of death; for surviving patients, the difference between diagnosis and 30/09/2023. |
| Five Year Survival | Binary Y/N |

**Supplementary Table 2 – Time in weeks between diagnosis and each clinical pathway milestone by rurality for each treatment regimen subgroup.**

| Time to Milestone (weeks)  Mean (SD) | Metro & Regional | Rural | p-value | Statistic |
| --- | --- | --- | --- | --- |
| *Radiation Therapy Only* | | | | |
| Surgery | 2.7 (1.6) | 3.2 (1.6) | **0.003** | *U* = 3192  *r* = 0.201 (medium) |
| Final Surgery | 3.2 (2.2) | 3.6 (2.2) | **0.029** | *U* = 3522  *r* = 0.146 (small) |
| Oncologist Referral | 5.3 (3.1) | 6.6 (3.3) | **0.004** | *U* = 3020  *r* = 0.192 (small) |
| Oncologist Consultation | 7.9 (3.0) | 8.4 (2.6) | 0.062 | - |
| TNM Staging | 7.6 (3.1) | 8.8 (3.5) | **0.006** | *U* = 3292  *r* = 0.184 (small) |
| MDT | 5.1 (2.1) | 6.8 (2.7) | **<0.001** | *U* = 2414  *r* = 0.321 (medium) |
| Radiation Therapy | 13.5 (3.9) | 14.6 (3.4) | **0.021** | *U* = 3398  *r* = 0.154 (small) |
| *Chemotherapy Only* | | | | |
| Surgery | 2.8 (1.5) | 3.3 (2.0) | 0.3 | - |
| Final Surgery | 3.2 (1.6) | 3.7 (2.6) | 0.7 | *-* |
| Oncologist Referral | 5.7 (3.5) | 6.3 (3.8) | 0.4 | - |
| Oncologist Consultation | 8.2 (3.4) | 8.8 (4.3) | 0.9 | - |
| TNM Staging | 7.3 (4.0) | 8.6 (8.5) | 0.9 | *-* |
| MDT | 4.6 (1.5) | 6.9 (3.2) | **<0.001** | *U* = 240  *r* = 0.415 (large) |
| Chemotherapy or Systemic Therapy | 12.8 (9.0) | 11.7 (3.4) | 0.3 | *-* |
| *Both adjuvant therapies* | | | | |
| Surgery | 2.4 (1.0) | 3.3 (1.6) | **<0.001** | *U* = 2062  *r* = 0.262 (medium) |
| Final Surgery | 2.8 (1.5) | 3.7 (2.2) | **0.020** | *U* = 2392  *r* = 0.178 (small) |
| Oncologist Referral | 5.8 (4.3) | 7.3 (3.2) | **<0.001** | *U* = 1684  *r* = 0.324 (medium) |
| Oncologist Consultation | 7.5 (4.0) | 7.9 (3.7) | 0.3 | *-* |
| TNM Staging | 8.4 (4.9) | 8.7 (7.5) | 0.2 | *-* |
| MDT | 6.1 (14.9) | 6.0 (2.3) | **<0.001** | *U* = 1858  *r* = 0.285 (medium) |
| Chemotherapy or Systemic Therapy | 9.6 (3.9) | 11.7 (3.6) | **<0.001** | *U* = 1802  *r* = 0.328 (medium) |
| Radiation Therapy | 31.2 (7.1) | 33.6 (7.2) | **0.033** | *U* = 2450  *r* = 0.162 (small) |
| *No adjuvant therapy* | | | | |
| Surgery | 2.8 (2.0) | 4.2 (2.0) | **<0.001** | *U* = 2598  *r* = 0.395 (medium) |
| Final Surgery | 3.4 (3.0) | 4.6 (2.5) | **<0.001** | *U* = 3012  *r* = 0.331 (medium) |
| Oncologist Referral | 5.8 (4.9) | 8.3 (4.2) | **<0.001** | *U* = 856  *r* = 0.418 (large) |
| Oncologist Consultation | 8.3 (5.2) | 9.6 (5.4) | 0.10 | *-* |
| TNM Staging | 6.8 (8.6) | 7.5 (5.7) | 0.072 | *-* |
| MDT | 5.0 (2.6) | 7.3 (2.8) | **<0.001** | *U* = 2150  *r* = 0.465 (large) |
